# Synthesized Heparan Sulfate Competitors Attenuate *Pseudomonas aeruginosa* Lung Infection

**DOI:** 10.3390/ijms19010207

**Published:** 2018-01-09

**Authors:** Nicola Ivan Lorè, Noemi Veraldi, Camilla Riva, Barbara Sipione, Lorenza Spagnuolo, Ida De Fino, Medede Melessike, Elisa Calzi, Alessandra Bragonzi, Annamaria Naggi, Cristina Cigana

**Affiliations:** 1 Division of Immunology, Transplantation and Infectious Diseases, IRCCS San Raffaele Scientific Institute, Milano 20132, Italy; riva.camilla@hsr.it (C.R.); sipione.barbara@hsr.it (B.S.); lorenza.spagnuolo@quintilesims.com (L.S.); defino.ida@hsr.it (I.D.F.); melessike.medede@hsr.it (M.M.); bragonzi.alessandra@hsr.it (A.B.); 2 Vita-Salute San Raffaele University, Milano 20132, Italy; 3 Istituto di Ricerche Chimiche e Biochimiche “G. Ronzoni”, Milano 20133, Italy; noemi.veraldi@gmail.com (N.V.); elisacalzi@libero.it (E.C.); naggi@ronzoni.it (A.N.)

**Keywords:** *Pseudomonas aeruginosa* infections, glycosaminoglycans, anti-inflammatory drugs, mouse models, chronic respiratory diseases

## Abstract

Several chronic respiratory diseases are characterized by recurrent and/or persistent infections, chronic inflammatory responses and tissue remodeling, including increased levels of glycosaminoglycans which are known structural components of the airways. Among glycosaminoglycans, heparan sulfate (HS) has been suggested to contribute to excessive inflammatory responses. Here, we aim at (i) investigating whether long-term infection by *Pseudomonas aeruginosa*, one of the most worrisome threat in chronic respiratory diseases, may impact HS levels, and (ii) exploring HS competitors as potential anti-inflammatory drugs during *P. aeruginosa* pneumonia. *P. aeruginosa* clinical strains and ad-hoc synthesized HS competitors were used in vitro and in murine models of lung infection. During long-term chronic *P. aeruginosa* colonization, infected mice showed higher heparin/HS levels, evaluated by high performance liquid chromatography-mass spectrometry after selective enzymatic digestion, compared to uninfected mice. Among HS competitors, an N-acetyl heparin and a glycol-split heparin dampened leukocyte recruitment and cytokine/chemokine production induced by acute and chronic *P. aeruginosa* pneumonia in mice. Furthermore, treatment with HS competitors reduced bacterial burden during chronic murine lung infection. In vitro, *P. aeruginosa* biofilm formation decreased upon treatment with HS competitors. Overall, these findings support further evaluation of HS competitors as a novel therapy to counteract inflammation and infection during *P. aeruginosa* pneumonia.

## 1. Introduction

Chronic respiratory diseases with different etiology, such as cystic fibrosis (CF), non-CF bronchiectasis, idiopathic pulmonary fibrosis (IPF) and advanced chronic obstructive pulmonary disease (COPD) show common traits such as recurrent and/or persistent infections, together with chronic inflammatory responses and immunopathology [1,2,3]. In particular, *Pseudomonas aeruginosa* infections are associated with an exaggerated inflammatory response, including neutrophil recruitment, and excessive tissue remodeling. The pathophysiological mechanisms underlying this immunopathological scenario in response to *P. aeruginosa* infections during chronic airway diseases remain to be deciphered.

The lung extracellular matrix represents a highly dynamic complex of fibrous proteins, glycoproteins, and proteoglycans, that composes the non-cellular aspect of tissues and varies in composition according to pathophysiological circumstances. In this context, glycosaminoglycans (GAGs) are long, linear, and heterogeneous polysaccharides formed by repetition of disaccharide units, that not only represent principal components of the extracellular matrix, but are also distributed in the subepithelial tissue, bronchial walls and airway secretions [4]. They include hyaluronic acid, dermatan sulfate (DS), keratan sulfate, heparin and chondroitin sulfate (CS), but the most abundant in the lung parenchyma is heparan sulfate (HS). HS binds several effector molecules, known to be involved in chronic respiratory disease such as COPD and CF. For example, the binding of chemokines to GAGs is thought to favor the generation of the chemotactic gradient [5] responsible for leukocyte recruitment to the site of infection/injury. In addition, HS can bind cytokines and chemokines, such as interferon-(IFN-)γ and interleukin-(IL-)8, protecting them from proteolytic degradation and, thus, increasing their activities [6,7]. Therefore, high neutrophil recruitment in the airways of patients with chronic respiratory diseases may be due not only to increased chemokines expression, but also to their increased stability and prolonged activity when they are bound to HS. This pathological scenario would finally include also tissue remodeling and fibrosis.

Reports indicate that not only the amounts, but also sulfation of GAGs are markedly increased in CF tissues. Secretion of HS is elevated in CF airways, potentially correlating with the exaggerated inflammatory response described in the CF lung [7]. HS levels are increased also in the bronchoalveolar lavage fluid (BALF) of COPD patients with bacterial infections and during exacerbations [8]. In addition, high levels and sulfation of HS have been described also in patients with IPF [9].

Taking into consideration both the increased levels of HS in the context of chronic respiratory diseases and their potential involvement in the pathogenesis, the disruption of the interaction between HS and cytokines/chemokines may impact the inflammatory response. Specific GAG mimetics have been used to target some of these interactions in in vitro models [10]. In this context, we recently tested and characterized several synthesized HS competitors, in particular chemically modified derivatives of heparin with attenuated anticoagulant activity. These HS competitors not only bind to cytokines/chemokines [10], as natural endogenous HS does, but also inhibit the activity of neutrophil elastase (NE), a protease involved in the progression of lung fibrosis and in the sustainment of the inflammatory response in chronic respiratory diseases.

To date, the relevance of HS during *P. aeruginosa* infections, that characterize many chronic respiratory diseases, has not yet been addressed. Here, we utilized mouse models of lung infection to (i) investigate whether chronic *P. aeruginosa* lung infection may impact HS composition, and (ii) explore the potential anti-inflammatory activity of synthesized HS competitors in vivo. We report that: (i) chronic *P. aeruginosa* lung infection increased the levels of specific HS disaccharide building blocks; (ii) two HS competitors, in particular an *N*-acetyl heparin and a glycol-split heparin, named respectively C23 and C3_gs20_, reduced the inflammatory response during acute and chronic *P. aeruginosa* lung infections, and decreased also the bacterial burden in a model of long-term chronic airways colonization.

## 2. Results

### 2.1. Evaluation of Specific HS Disaccharides and Their Levels in a Murine Model of Chronic P. aeruginosa Airways Infection

**Figure 1:**
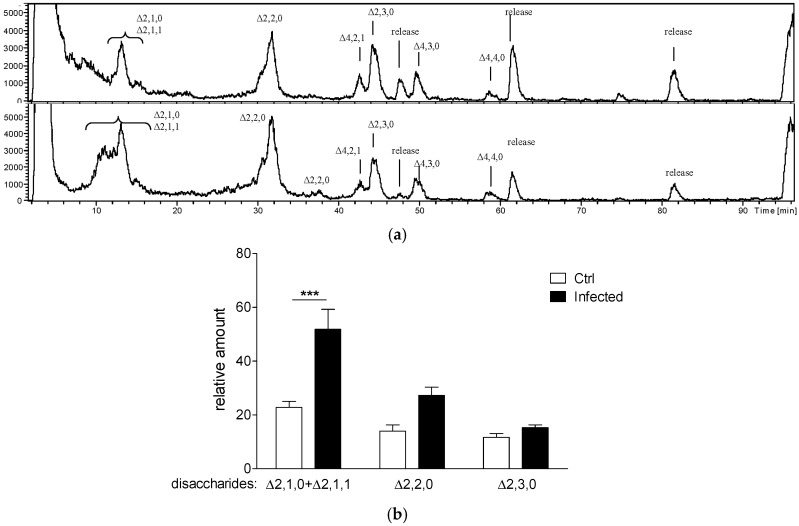
Disaccharide products of the digestion of heparin/HS from murine lungs after chronic *P. aeruginosa* infection. C57Bl/6NcrlBR mice were intratracheally injected with 1–2 × 10^6^ colony forming units (CFUs) of *P. aeruginosa* isolate AA43 embedded in agar-beads (Infected) or with sterile agar-beads (Ctrl). After 28 days, lungs were perfused, recovered, homogenized and separated into pellets and supernatants. After removal of proteins, lipids and DNA, the presence of GAGs was verified by NMR. Samples were digested with a cocktail of heparin lyases to selectively degrade heparin/HS and recovered digestion products were desalted and finally analyzed by HPLC-MS. (**a**) Base Peak Chromatogram of digestion products from the pellet of one Ctrl uninfected (**upper** panel) and one infected (**lower** panel) lung homogenate from C57Bl/6NcrlBR mice. The unsaturated bond of the terminal uronic acid is indicated by Δ, and the number of monomers, the number of sulfates and the number of acetyls are reported; (**b**) The graph shows the amount of each disaccharide species detected in the whole lung of an infected and an uninfected Ctrl mouse; 100% is considered the sum of peak areas of one whole lung from infected mouse lungs containing the highest amount of disaccharides. The data are pooled from at least two independent experiments (*n* = 6–15). Data are the mean ± standard error of the mean (SEM) of at least three samples per type which have been processed independently. Statistical significance is indicated: *** *p* < 0.001.

Taking into consideration the increased levels of GAGs in the lungs of mice chronically infected with *P. aeruginosa* for 28 days [11], we evaluated GAG species in this mouse model of chronic airway infection. C57Bl/6NcrlBR mice were inoculated with *P. aeruginosa* AA43 isolate embedded in agar-beads and with sterile agar beads (Ctrl group). After 28 days murine lungs were homogenized, centrifuged and supernatants were separated from pellets to distinguish released GAGs from those present as structural components of the extracellular matrix, respectively. Only lungs from mice that were still infected at 28 days post-challenge were analysed for their GAG content by selective enzymatic digestion [12,13]. High performance liquid chromatography-mass spectrometry (HPLC-MS) profiles of digestion products showed the presence of oligosaccharides from heparin/HS (Figure 1a and Supplementary Figure S1b), but not from other GAGs such as CS or DS. The amount of CS/DS was probably under the limit of detection. Digestion with heparinases lyases produced mainly disaccharides and traces of tetrasaccharides and hexasaccharides. It is known that the presence of the 3-*O*-sulfated glucosamine renders the glycosidic bond between *N*-acetylated, 6-*O*-sulfated glucosamine and the unsulfated glucuronic acid impervious to the action of heparinases [14]. Indeed, we detected tetrasaccharides probably bearing the trisulfated glucosamine residue like Δ4,4,0, but also other tetrasaccharides, i.e., Δ4,3,0 and Δ4,2,1, probably due to a limited efficiency of the enzymatic digestion, despite the excess of enzymes. Thus, we considered the disaccharidic composition, that represents more than the 90% of the digestion products in both infected and uninfected mice. The overall composition of digestion products indicated the presence of HS-like structure rather than heparin, due to the scarce presence of the trisulfated disaccharide (Δ2,3,0) and the high presence of monosulfated or monoacetylated disaccharides (Δ2,1,0 and Δ2,1,1). Indeed, comparing the integrals of HPLC peaks of disaccharides to the sum of the relative integrals (Figure 1b), an increase in digestion products in infected mice when compared to control uninfected mice was observed, with the prevalence of the monosulfated and disulfated disaccharides over the other species detected. More in details, this difference was observed in pellets of lung homogenates (Supplementary Figure S1a), indicating that chronic *P. aeruginosa* lung infection increased the levels of structural HS. Differently, no significant differences were found in the supernatants of infected lungs compared to those of Ctrl uninfected lungs (Supplementary Figure S1a,b).

### 2.2. Structure and Efficacy of Synthesized HS Competitors in the Mouse Model of Acute P. aeruginosa Airways Infection

**Figure 2:**
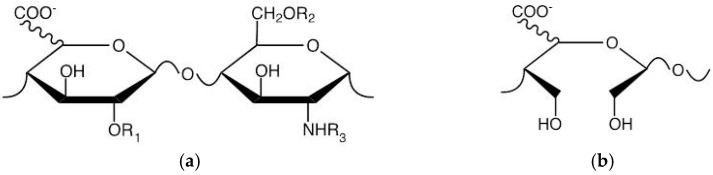
Structures and characteristics of synthesized HS competitors. The repeating disaccharide unit of compounds (R_1_ and R_2_ = H/SO_3_^−^, R_3_ = H/SO_3_^−^/COCH_3_) is shown. The uronic acid is predominantly in the form l-iduronic acid (l-IdoA and l-IdoA-2-*O*-sulfate; ~80%) with d-glucuronic acid (d-GlcA; ~20%) making up the remainder. (**a**) Structure of the canonical disaccharide building block of heparin; (**b**) The glycol-split uronic acid residue present in compounds C3_gs20_, MMW C3_gs90_ and MMW C3_gs45_.

We designed and characterized specific heparin derivatives to generate compounds able to act as competitors of endogenous HS in the murine lung. In particular, these HS competitors have been prepared starting from unmodified pig mucosal heparin (PMH) (Table 1) by targeted chemical modification able to strongly diminish/abolish its anticoagulant activity while maintaining the ability to interact with other proteins [10]. The first set of HS competitors included glycol-split heparin C3_gs20_ and *N*-acetyl heparin C23, prepared as described previously [15]. In Figure 2, the major repeating disaccharide unit of heparin derivatives and the structure of the glycol-split uronic acid are shown.

Size is an important parameter that can influence the binding of HS competitors to proteins, especially the minimum length that is required to establish an interaction. For the second set of HS competitors to be tested, two structurally low molecular weight (LMW) analogues of C3_gs20_ and C23 have been prepared by controlled reductive deamination, in order to improve the bio-availability of compounds and to verify the influence of dimensions on the activity of derivatives. In addition, the second set of synthesized HS competitors included compounds MMW C3_gs90_ and MMW C3_gs45_, that have been partially desulfated in order to increase the percentage of non-sulfated uronic acids reactive to the glycol-splitting modification to obtain a higher percentage of flexible joints along the chains. The MW of the first and second sets of compounds prepared (Table 1) ranged from 8 to 17.2 kDa, assuring an interaction with both IL-8 and human NE (12–14-mers for NE and 18-mer for IL-8) [16,17] which are known to be relevant to chronic inflammatory conditions. Notably, under controlled periodate oxidation conditions it is possible to limit the cleavage of glycosidic bonds without significant depolymerization of the HS competitors, as in the case of C3_gs20_. Nevertheless, the combination of desulfation followed by glycol-splitting led to decrease in the MW of MMW C3_gs45_ and MMW C3_gs90_ proportionally to the increase of glycol-splitting, as expected.

**Figure 3:**
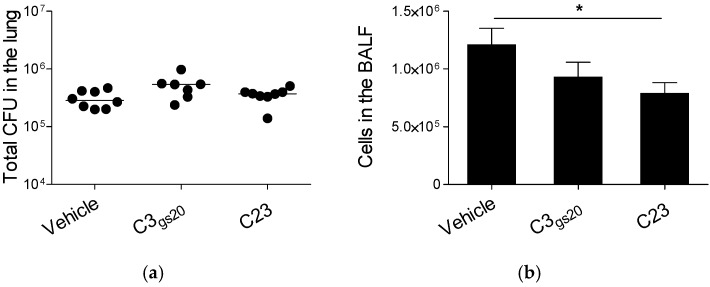

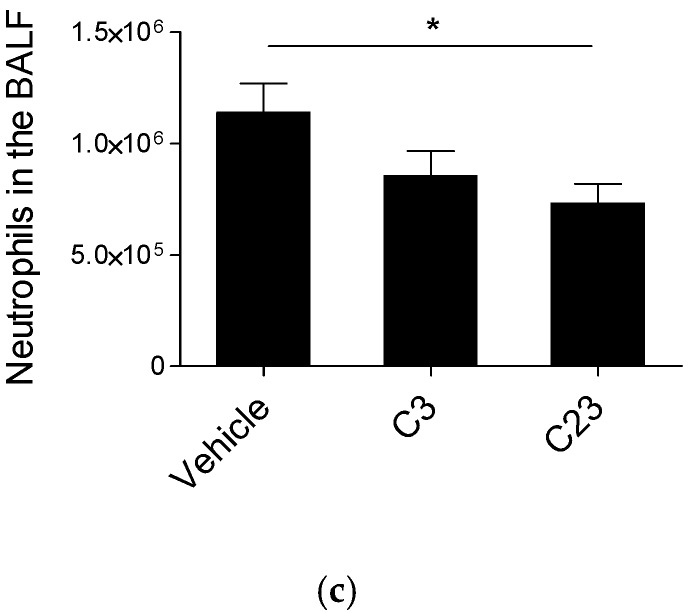
Modulation of the host response by synthesized HS competitors in a mouse model of acute *P. aeruginosa* lung infection. C57Bl/6NcrlBR mice were intratracheally injected with 5 × 10^6^ CFUs of the highly virulent *P. aeruginosa* isolate AA2. Mice were subcutaneously treated with HS competitors (30 mg/kg) or their vehicle two hours before and two hours after the challenge and sacrificed 6 h post-infection. BALF and lung were recovered. (**a**) Total CFUs in the lungs were evaluated; (**b**) Total cell and (**c**) neutrophil recruitment was analyzed in BALF. The data are pooled from at least two independent experiments (*n* = 7–8). CFUs in individual mice are represented as dots and horizontal lines represent median values. Cells are represented as mean ± SEM. Statistical significance is indicated: * *p* < 0.05.

Heparin derivatives have been shown to exert beneficial effects in inflammation [18]. We thus evaluated the impact of C3_gs20_ and C23 on leukocyte recruitment in the murine BALF following acute *P. aeruginosa* lung infection. Mice were infected with the *P. aeruginosa* AA2 isolate [11,19], known to be highly virulent, and treated subcutaneously with these compounds (30 mg/kg). We found that neither C23 nor C3_gs20_ affected the lung bacterial burden when compared to the vehicle (Figure 3a). C23 significantly reduced the number of total leukocytes in comparison to the vehicle (Figure 3b) and in particular neutrophils (Figure 3c) in the BALF, although the neutrophil percentages were not significantly different between mice treated with the compound and those treated with the vehicle (C23 vs. vehicle *p* = 0.057). Differently, C3_gs20_ had only moderate inhibitory effects on leukocyte recruitment. C23 significantly reduced also IL-6, IL-12 (p40), granulocyte colony-stimulating factor (G-CSF) and monocyte chemoattractant protein-1 (MCP-1) when compared to the vehicle (Table 2). C3_gs20_ reduced inflammatory mediators but at lower extent and with a statistically significant difference only for IL-6 levels (Table 2). Differently, when the second set of HS competitors, including LMW C3_gs20_, LMW C23, MMW C3_gs90_ and MMW C3_gs45_, were tested in this murine model, they did not affect either the bacterial burden or the recruitment of leukocytes, including neutrophils, in the BALF (Supplementary Figure S2).

Overall, these results indicate C23 and C3_gs20_ as the most promising anti-inflammatory compounds in the mouse model of acute *P. aeruginosa* lung infection.

### 2.3. Efficacy of Synthesized HS Competitors in Mouse Models of Chronic P. aeruginosa Airways Infection

**Figure 4:**
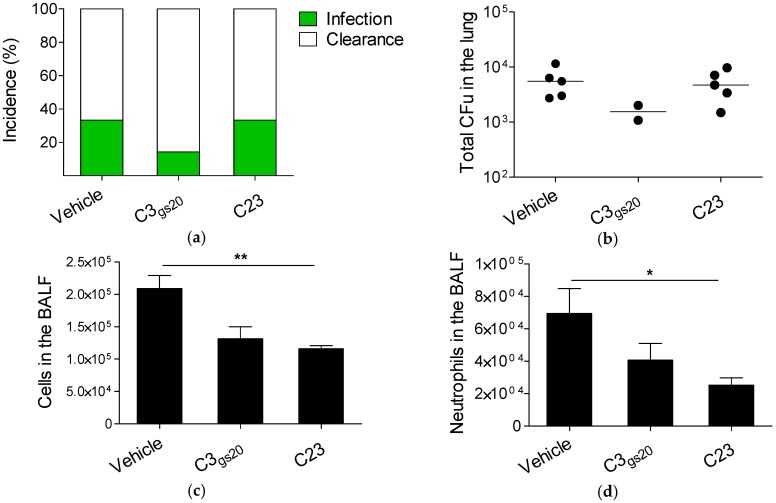
Modulation of the host response by synthesized HS competitors in a mouse model of chronic *P. aeruginosa* lung infection (14 days). C57Bl/6NcrlBR mice were intratracheally injected with 1–2 × 10^6^ CFUs of the *P. aeruginosa* isolate AA43 embedded in agar-beads. Mice were treated subcutaneously with HS competitors (30 mg/kg) or vehicle every day starting from the day of infection for 14 days. At the sacrifice, BALF and lung were recovered. (**a**) Bacterial clearance (white) and incidence of colonization (green) were determined; (**b**) CFUs were evaluated in total lung of mice still infected at the sacrifice. Total cell (**c**) and neutrophil (**d**) recruitment was analyzed in BALF. The data are pooled from two independent experiments (*n* = 14–15). CFUs in individual mice are represented as dots and horizontal lines represent median values. Cells are represented as mean ± SEM. Statistical significance is indicated: * *p* < 0.05, ** *p* < 0.01.

Next, we tested the ability of HS competitors to reduce inflammation during the development and the course of chronic *P. aeruginosa* lung infection. First, we evaluated the effect of C3_gs20_ and C23 in an agar-beads mouse model of chronic lung infection running for 14 days. C57Bl/6NcrlBR mice were chronically infected with *P. aeruginosa* AA43 isolate, known to persist in the lung with an incidence of colonization around 30–40% [11], and treated subcutaneously with C3_gs20_ and C23 starting from the day of infection. We found that the incidence of chronic lung colonization was reduced in mice infected with C3_gs20_ when compared to those treated with the vehicle (13% for C3_gs20_ and 33% for C23 vs. 33% for vehicle; Figure 4a), although this difference did not reach statistical significance. We also observed a trend to a decrease in lung CFUs in mice treated with C3_gs20_ in comparison to vehicle (Figure 4b). In addition, C23 significantly reduced the recruitment of total leukocytes, including neutrophils, in comparison to vehicle (Figure 4c,d). A similar trend was observed also for C3_gs20_.

**Figure 5:**
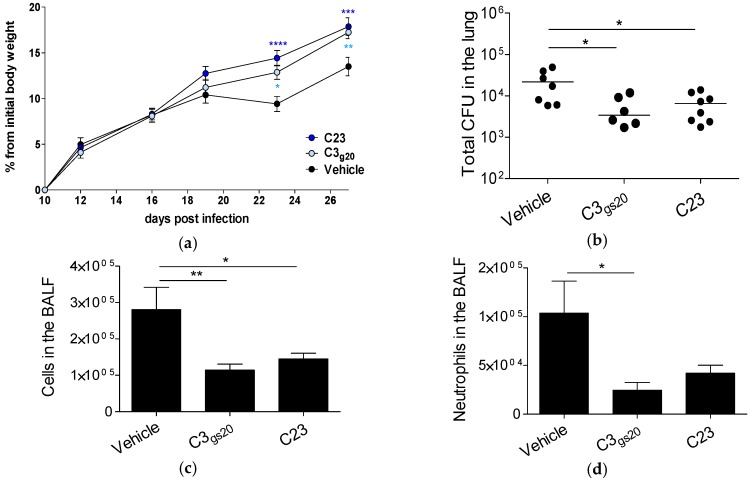
Modulation of the host response by synthesized HS competitors in a mouse model of long-term chronic *P. aeruginosa* lung infection (28 days). C57Bl/6NcrlBR mice were intratracheally injected with 1–2 × 10^6^ CFUs of the *P. aeruginosa* isolate AA43 embedded in agar-beads. Mice were treated subcutaneously with HS competitors (30 mg/kg) or vehicle every day starting from ten days post-infection. At the sacrifice, BALF and lung were recovered. (**a**) Changes from initial body weight were calculated for each group of mice at regular intervals. (**b**) Total CFUs in the lungs were evaluated. (**c**) Total cell and (**d**) neutrophil recruitment was analyzed in BALF. The data are pooled from at least two independent experiments (*n* = 20–26). CFUs in individual mice are represented as dots and horizontal lines represent median values. The other parameters are represented as mean ± SEM. Statistical significance is indicated: * *p* < 0.05, ** *p* < 0.01, *** *p* < 0.001, **** *p* < 0.0001.

We then investigated whether C3_gs20_ and C23 also impacted an established chronic *P. aeruginosa* lung infection. Thus, we extended the *P. aeruginosa* chronic infection for 28 days and started the treatment with HS competitors and vehicle after ten days from the infection, once chronic infection is well-established [20]. We previously showed that this mouse model of *P. aeruginosa* persistence is highly stable in terms of incidence of colonization and bacterial burden up to three months, and reproduces detectable chronic inflammation and tissue damage for long-term [11,20]. By using this schedule of treatment, the incidence of chronic *P. aeruginosa* lung colonization was not affected by HS competitors and remained stable (percentages of mice still colonized after 28 days: vehicle, 30.4%; C3_gs20_, 30%; C23, 30.8%). However, C3_gs20_ and C23 significantly increased murine body weights when compared with the vehicle, indicating that they promoted a better health status (Figure 5a). In addition, C3_gs20_ and C23 significantly decreased the bacterial burden in the lung in comparison to the vehicle (Figure 5b). C3_gs20_ significantly decreased the infiltration of leukocytes, including neutrophils, in the BALF (Figure 5c,d), and the levels of inflammatory mediators, such as IL-1β, IL-12p70, IL-17A, G-CSF and KC in comparison to the vehicle (Table 3). C23 showed a similar trend, although only the differences of leukocyte infiltration and IL-17A level reached a statistical significance (Figure 5c and Table 3). Other markers were not affected by these compounds as shown in Table 3.

When we tested the impact of other HS competitors on the host response in this mouse model of chronic lung infection, we found that neither MMW C3_gs90_ nor MMW C3_gs45_ affected either bacterial burden or leukocytes recruitment (Supplementary Figure S3).

Overall these results indicated that C3_gs20_ and C23 have a dual effect: on inflammation, containing leukocyte recruitment in the site of infection, and on infection, reducing the bacterial load.

**Figure 6:**
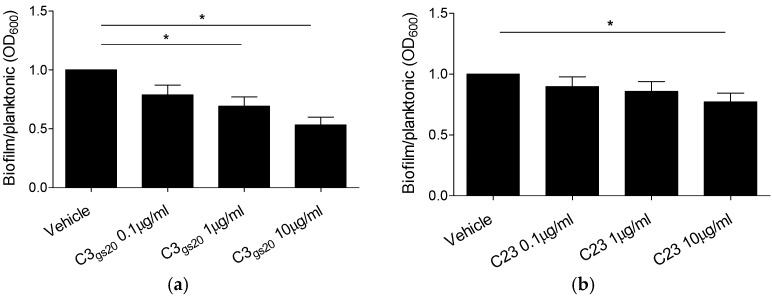
Effect of synthesized HS competitors on *P. aeruginosa* biofilm formation. *P. aeruginosa* strain AA43 was grown for 24 h at 37 °C either in the absence or presence of different concentrations of C3_gs20_ (**a**) and C23 (**b**). Biofilm biomass was quantified by staining with crystal violet and absorbance measurements at OD_600_. Absorbance of planktonic bacteria in the culture medium was measured at OD_600_. Results are expressed as the ratio between biofilm absorbance and planktonic bacteria absorbance normalized on the value obtained for AA43 treated with isotonic saline (vehicle). The data derive from three independent experiments in triplicate. Values represent the mean ± SEM. Statistical significance is indicated: * *p* < 0.05.

Taking into consideration the effect of C3_gs20_ and C23 on *P. aeruginosa* bacterial burden in the mouse model of chronic *P. aeruginosa* infection, we asked whether C3_gs20_ and C23 could have any anti-bacterial activity. We did not find any change in minimum inhibitory concentrations after challenge with up to 512 µg/mL. When we investigated the effect on biofilm formation, both C3_gs20_ and C23 induced a statistically significant reduction of AA43 sessile fraction in a dose-dependent fashion (Figure 6). These data suggest an inhibitory effect exerted by C3_gs20_ and C23 on *P. aeruginosa* biofilm formation.

## 3. Discussion

Increased levels of GAGs including HS, the most abundant in the lung parenchyma, are common to several chronic respiratory diseases, including CF, COPD and IPF [1,2,5]. Whether *P. aeruginosa* infections may contribute to the increase of HS is still an open question. Clinical data are difficult to interpret due to the large number of confounding variables. Here, using mouse models of lung infection, we demonstrated that long-term chronic *P. aeruginosa* lung infection induces structural changes in HS as shown by increased amounts of specific HS disaccharide building blocks. In addition, our results indicate that two synthesized HS competitors, in particular *N*-acetyl heparin and glycol-split heparin, named respectively C23 and C3_gs20_, competing with endogenous HS, dampen the inflammatory response induced by *P. aeruginosa* and reduce the bacterial burden.

To our knowledge, despite several reports indicating increased levels of highly sulfated HS in the airways of patients affected by chronic respiratory diseases, there are no studies analyzing specifically the impact of bacterial infections on HS levels and composition in the lung. We previously demonstrated that *P. aeruginosa* persistence was associated with a progressive increase of sulfated GAGs in murine lungs [11]. Here, we focused on HS composition and found that long-term chronic *P. aeruginosa* lung colonization led to an increase of specific HS disaccharides, in particular mono- and di-sulfated ones, in the pellet of murine lungs. This finding suggests that *P. aeruginosa* infection increases mainly structural HS, rather than contributing to released HS. In this regard, HS has been detected also in the BALF in COPD patients [8] and in the sputum sol of patients with bronchiectasis [21] indicating HS degradation. However, no reports indicate a correlation between HS levels in airway secretions and bacterial infection. Future clinical studies should determine a potential correlation between bacterial infections/colonization, including by *P. aeruginosa*, and the levels and sulfation of HS in the lungs of patients with chronic respiratory diseases. In addition, we analyzed the HS composition in the lung of C57Bl/6NcrlBR mice. Since the murine genetic background determines infection outcomes [22,23,24], it would be interesting to clarify whether it can also affect the composition of the extracellular matrix by using different inbred and outbred murine lines.

Besides maintaining lung tissue structure, HS may modulate the behavior of cells by binding growth factors and by interacting with cell surface receptors [4,25]. In addition, it can interact with proteases and cytokines/chemokines impacting on immunopathology. Taking into consideration these evidences and our findings on the increase of HS during chronic *P. aeruginosa* lung infection, we explored a HS inhibitory strategy in mouse models of infection. We chose to chemically modify heparin to synthesize competitors of the endogenous HS, thanks to its structural similarity to HS and its commercial availability; moreover, it is commonly used in hospitals as an anticoagulant drug and considered to be safe. Recently, these synthesized HS competitors with low or absent anticoagulant activity have been shown to bind to IL-8 and TNF-α and to inhibit the activity of NE in vitro [10], suggesting that these compounds could preserve the connective tissues and limit inflammation in vivo. When tested in murine models, C23 and C3_gs20_ reduced neutrophilic recruitment and cytokines/chemokines production both in acute and chronic *P. aeruginosa* lung infection. These results support the finding that HS binding to chemokines may contribute to leukocyte recruitment in the site of infection/injury by generating the chemotactic gradient as well as increasing pro-inflammatory activities of chemokines by protecting them from proteolytic degradation [5,6,7]. It can be hypothesized that synthesized HS competitors may reduce levels of endogenous HS, thus reducing the chemotactic gradient. In this regard, a HS reduction secondary to decreased inflammation and infection may be plausible after long-term chronic lung infection. However, it would be unlikely that these compounds can directly reduce HS, taking into account their effects after short-term acute *P. aeruginosa* infection. Differently, we speculate that synthesized HS competitors could have competed with endogenous HS for the binding to cytokines/chemokines, thus leading to their degradation/elimination. Further studies to elucidate these mechanisms are necessary, in particular to evaluate the amount of chemokines still bound to endogenous HS during treatment with synthesized HS competitors in vivo. Anyhow, the reduction of neutrophilic recruitment could lead to a decreased immunopathology by itself, since it implies lower levels of neutrophil proteases, including NE. In addition, taking into consideration that HS competitors inhibit NE activity [10], reduced tissue remodeling and fibrosis could be expected. In fact, NE, a protease described as abundant in the airways of patients affected by chronic respiratory diseases [26], degrades several components of the extracellular matrix, including elastin [27], thus contributing to the tissue remodeling and fibrosis [4], and ultimately aggravating the immunopathology. In this context, several attempts to inhibit NE have been carried out, also in clinical studies on patients with respiratory diseases, although with contrasting results [26].

One of the off-target effects of anti-inflammatory treatments is the impairment of the host defense that can lead to the exacerbation of the infection [20,28,29,30]. However, we did not observe an increase in *P. aeruginosa* burden in the model of acute lung infection. Next, we set-up two schedules of treatment in the mouse model of chronic lung infection. In the 14-day-lasting model of chronic lung colonization, mice had been treated starting from the day of infection. In such a model, synthesized HS competitors could interfere in particular with the early immune response, thus impacting the development and the progression of chronic lung infection. Differently, in the 28-day-lasting model of chronic lung colonization, mice were treated starting from ten days post-infection to evaluate effects of HS competitors on well-established chronic lung infection. In both schedules, we adopted the *P. aeruginosa* AA43 isolate, which can establish chronic infection in C57Bl/6NcrlBR mice with an incidence of colonization around 30–40% [11] starting from seven days post-infection. This choice is fundamental when the aim is determining the impact of anti-inflammatory treatment on the host defense, since an increase in the number of mice still colonized after long-term or in *P. aeruginosa* load could be observed. For instance, using this isolate we recently demonstrated that the IL-17A pathway impairment increased both the incidence of colonization and bacterial burden [20]. Unexpectedly, C3_gs20_ and C23 impacted *P. aeruginosa* infection and, in particular, they reduced the bacterial burden in murine lungs after long-term chronic lung infection.

When administered in different schedules of *P. aeruginosa* lung infection and treatment, C3_gs20_ and C23 showed slight differences: C23 was more potent in inhibiting the inflammatory response in the model of acute lung infection, while C3_gs20_ was more effective in the model of long-term chronic infection, where it decreased the bacterial burden, and it was more potent in hampering in vitro *P. aeruginosa* biofilm formation. We may speculate that these different activities may be addressed to the structures of synthesized HS competitors. Indeed, C23 is a *N*-acetyl heparin, with charge density and distribution similar to that of HS. Therefore, it is potentially able to mimic HS in its interactions and to compete by sequestering cytokines/chemokines from endogenous HS, thus rendering them more susceptible to degradation. Differently from C23, C3_gs20_ maintains the negative charge density of PMH and displays chain flexibility thanks to the glycol-split modification. In this context, its more potent anti-biofilm activity could be due to several interactions: (i) because of its negative charges, it could bind Ca^++^, known to form a bridge between polyanionic alginate molecules that stabilizes biofilms [31]; (ii) it could interact with biofilm polysaccharides, impeding their binding; (iii) it could counteract the binding between single *P. aeruginosa* cells. However, there could be other explanations to the decrease of *P. aeruginosa* burden after long-term chronic lung colonization following treatment with C23, and in particular C3_gs20_. In fact, HS has been found to be a cell receptor for *P. aeruginosa* and a binding site for bacterial flagella, and this could suggest another role during infection [32,33]. Further studies based on additional structural modifications of these compounds could provide new insights into their biological activities during host–pathogen interplay. Moreover, the reduction of the bacterial burden by HS competitors during *P. aeruginosa* pneumonia suggests that the potential synergy between these compounds and antibiotics currently used in clinics should be investigated.

## 4. Materials and Methods

### 4.1. Ethics Statement

Animal studies strictly followed the Italian Ministry of Health guidelines for the use and care of experimental animals. This study was performed following protocols approved by the Institutional Animal Care and Use Committee (IACUC, protocols #502 of 14 July 2011 and #812 of 28 July 2016) of the San Raffaele Scientific Institute (Milan, Italy). Research with *P. aeruginosa* clinical strains has been approved by the responsible physician at the CF Center at Hannover Medical School, Germany. Additional information is available in the Supplementary Materials.

### 4.2. Bacterial Strains

Sequential *P. aeruginosa* isolates (AA2 and AA43) were recovered from CF patients and previously characterized for genotypic and phenotypic traits, and virulence [19,34,35]. AA2, expressing several virulence factors, including swimming motility, twitching motility and protease secretion, was isolated at the onset of chronic infection. Differently, AA43, a variant with adaptive phenotypes, including mucoidy, absence of swimming motility, low twitching motility and production of protease, was collected after seven years of chronic colonization. AA2 has previously been shown to induce high in vitro and in vivo acute virulence, while AA43 has been shown to be attenuated, but capable of developing chronic lung infection [11,19,36].

### 4.3. Mouse Strain

C57Bl/6NcrlBR (Charles River, Calco, Lecco, Italy), 8 to 10 weeks old, were maintained in specific pathogen-free conditions at the San Raffaele Scientific Institute (Milan, Italy).

### 4.4. HS Competitors Synthesis and Characterization

Compounds are HS competitors obtained by chemical modification of PMH in order to reduce the anticoagulant activity whilst maintaining the anti-inflammatory potential. In particular, the *N*-acetyl heparin C23 and glycol-split heparin derivatives C3_gs20_, MMW C3_gs90_ and MMW C3_gs45_, were generated as previously described [15,37] and characterized by ^13^C-NMR [10] (Supplementary Figure S4a). Additional information is available in the Supplementary Materials. In addition, two LMW variants of C23 and C3_gs20_ were produced by depolymerization of PMH through reductive deamination with nitrous acid using a NaNO_2_/heparin ratio of 1:7, as explained in detail in the Supplementary Materials, followed by the same modifications introduced on full-length derivatives. These LMW were characterized by HSQC-NMR (Supplementary Figure S4b). The average MWs were determined at a concentration of 5 mg/mL employing Viscotek HP-SEC-TDA as previously described [38].

### 4.5. Mouse Models of Acute and Chronic P. aeruginosa Infection

In the acute infection model, mice were injected intratracheally with 5 × 10^6^ CFUs of planktonic *P. aeruginosa* AA2 isolate, following established procedures [11,19,39]. Mice were treated subcutaneously with 30 mg/kg of synthesized HS competitors [40,41,42] or with vehicle (isotonic saline) two hours before and two hours after infection, and sacrificed six hours post-infection.

In the chronic infection model, mice were injected intratracheally with 1–2 × 10^6^ CFUs of *P. aeruginosa* AA43 isolate, embedded in agar beads, following established procedures [11,30,39]. The bacterial load was previously set-up as the minimum inoculum to establish chronic infection [43]. Another group of mice (Ctrl) was intratracheally injected with sterile beads. For the analysis of HS/heparin levels, one batch of infected mice and the Ctrl group were sacrificed after 28 days from the infection, lungs were perfused with isotonic saline to avoid the contamination by circulating HS/heparin, recovered, homogenized and centrifuged. Pellets and supernatants were separated and lyophilized. For the analysis of HS competitor efficacy, one batch of infected mice was treated once a day as described above starting from the day of infection and sacrificed 14 days post-infection. Another batch of mice was treated once a day as described above starting from ten days post-infection and sacrificed 28 days post-infection.

BALF and lung were recovered and processed, and total/differential cell count and bacterial burden evaluated, as previously described [39]. Further information on the procedures are present in the Supplementary Materials.

### 4.6. HS/heparin Analysis in Murine Lungs

Samples were defatted by washing with chloroform/methanol, then with ethyl ether and freeze-dried, recovered in dPBS with 2 mM CaCl_2_ and subjected first to proteolytic cleavage with Proteinase K at 55 °C for 48 h, then to DNase I digestion at 37 °C for 48 h. After boiling for 10 min to stop the reaction, samples were filtered on 0.2 μm filters, then purified by 3 kDa ultrafiltration to remove digestion fragments. Purified samples were analyzed by ^1^H-NMR and eventually subjected to a second protease digestion. Digestion of CS by Chondroitinase ABC was carried out in a 50 mM sodium acetate/phosphate buffer (1:1 *v*/*v*), pH 8 at 37 °C for 48 h. After inactivation by boiling for 10 min samples were filtered onto 0.45 μm cut-off filters.

Digestion of heparin and HS with a cocktail of heparin lyases I–II-III (Grampian Enzymes, Aberdeen, UK), was carried out at 37 °C for 48 h, then stopped by boiling for 10 min followed by 0.2 μm filtration. Products were recovered by 3 kDa ultrafiltration, desalted and lyophilized. 100 µL sample solution was prepared for HPLC-MS analysis on a LC system coupled with an ESI-Q-TOF mass-spectrometer (micrOTOFq, Bruker Daltonics, Bremen, Germany). The chromatographic separation was performed using a Kinetex C18 analytical column (100 × 2.1 mm, 2.6 μm particle size, Phenomenex, Torrance, CA, USA) with Security Guard Cartridges Gemini C18 (4 × 2.0 mm, Phenomenex). A binary solvent system was used for gradient elution at 0.1 ml/min of solvent A (10 mM DBA, 10 mM CH_3_COOH in water) and solvent B (10 mM DBA and 10 mM CH_3_COOH in methanol): t 0′ 10%B, t 35′ 40%B, t 85′ 50%B, t 88′ 90%B, t 95′ 10%B. Disaccharide standards were purchased from Iduron, Manchester, UK. Data were processed by the DataAnalysis software (HyStar Compass, version 3.2, Bruker Daltonics).

### 4.7. Evaluation of Cytokines/Chemokines

Cytokines/chemokines and growth factors were measured by Bioplex in the lung homogenates, according to the manufacturer’s instructions [11].

### 4.8. Evaluation of P. aeruginosa Biofilm Formation

Biofilm production was evaluated using the method of staining with crystal violet, as previously described [44]. AA43 was grown for 24 h at 37 °C either in the absence or presence of different concentrations of C3_gs20_ and C23 (10 µg/mL, 1 µg/mL and 0.1 µg/mL). Biofilm biomass was quantified by staining with crystal violet and absorbance measurements at OD_600_. Absorbance of planktonic bacteria in the culture medium was measured at OD_600_. Results are expressed as the ratio between biofilm absorbance and planktonic bacteria absorbance normalized on the value obtained for AA43 treated with the vehicle. Further details can be found in the Supplementary Materials.

### 4.9. Statistics

Statistics were performed with GraphPad Prism. Data analysis was performed using one-way ANOVA followed by Dunnett’s analysis to correct for multiple comparisons for CFU counts, cellular counts and cytokine/chemokines quantification. Incidences of chronic colonization were compared using Fisher exact test. Two-way ANOVA with Bonferroni’s Multiple Comparison test was used to compare changes in body weight. *p* <0.05 was considered significant.

## 5. Conclusions

In conclusion, our study shows that chronic *P. aeruginosa* lung infection increased HS levels in the murine lungs, and that interfering with this phenomenon dampened the inflammatory response. We indeed demonstrated that synthesized HS competitors decreased leukocyte recruitment and cytokine/chemokine production both during acute *P. aeruginosa* infection, and during the development and the course of chronic lung infection in mice. In addition, these compounds reduced *P. aeruginosa* infection in the agar-beads mouse model. Overall, these data support further evaluation of HS competitors as novel therapeutic molecules to counteract excessive inflammation induced by *P. aeruginosa* pulmonary infections in patients affected by chronic respiratory diseases.

## Figures and Tables

**Table 1:** Structural characteristics of synthesized HS competitors originating from PMH (MW 20 kDa, %NAc 15).

HS Competitors	MW (kDa)	*N*-acetyl (%)	Glycol-Split (%)
C23	17.2	100	0
C3_gs20_	16.5	14.6	20
LMW C23	8	91	0
LMW C3_gs20_	8	14.6	18
MMW C3_gs45_	12.6	14.6	45
MMW C3_gs90_	9.6	14.6	90

The average molecular weight (MW), percentage of *N*-acetyl substitution in glucosamine residues and percentage of glycol-split (gs) uronate residues (cleavage by periodate oxidation of vicinal diols in unsubstituted d-GlcA and l-IdoA residues) is reported. Samples MMW C3_gs90_ and MMW C3_gs45_ have been partially desulfated in order to increase the percentage of uronic acids reactive for the glycol-splitting modification. Samples LMW C23 and LMW C3_gs20_ have been obtained by controlled reductive deamination of PMH. LMW, low MW; MMW, medium MW.

**Table 2:** Levels of cytokines and chemokines in murine lungs during acute *P. aeruginosa* lung infection (6 h) and subcutaneous treatment with C3_gs20_ or C23 (30 mg/kg).

Cytokine/Chemokine	Level (pg/500 µg Lung)			*p* Value	
	Vehicle	C3_gs20_	C23	C3_gs20_ vs. Vehicle	C23 vs. Vehicle
IL-4	3.31 ± 0.27	3.54 ± 0.19	3.37 ± 0.63	ns	ns
IL-6	232.9 ± 8.53	176.9 ± 15.95	159.7 ± 7.14	**	***
IL-12p40	10.97 ± 0.79	9.21 ± 0.76	7.86 ± 0.68	ns	*
IL-12p70	83.98 ± 3.46	93.98 ± 9.67	87.04 ± 6.45	ns	ns
IL-13	124.68 ± 8.49	140.26 ± 6.32	114.37 ± 5.33	ns	ns
IL-17A	6.77 ± 0.78	6.85 ± 0.21	6.66 ± 0.99	ns	ns
Eotaxin	353.62 ± 22.52	450.79 ± 33.85	341.3 ± 71.56	ns	ns
G-CSF	542 ± 10.56	465.8 ± 37.58	426.9 ± 36.53	ns	*
IFN-γ	538.19 ± 15.12	525.13 ± 72.72	462.34 ± 54.04	ns	ns
MCP-1	1327 ± 89.7	1239 ± 111.1	864.2 ± 118.6	ns	*
MIP-1β	1302.61 ± 124.2	1308.63 ± 142.3	1003.01 ± 191.9	ns	ns
RANTES	195.83 ± 19.19	247.57 ± 39.04	248.55 ± 46.88	ns	ns

Data are expressed as mean ± SEM. Statistical significance is indicated: * *p* < 0.05, ** *p* < 0.01, *** *p* < 0.001. ns: not significant. MIP-1β, macrophage inflammatory protein-1β; RANTES, regulated on activation normal T expressed and secreted.

**Table 3:** Levels of cytokines and chemokines in murine lungs during chronic *P. aeruginosa* lung infection (28 days) and subcutaneous treatment with C3_gs20_ or C23 (30 mg/kg).

Cytokine/Chemokine	Level (pg/500 µg Lung)			*p* Value	
	Vehicle	C3_gs20_	C23	C3_gs20_ vs. Vehicle	C23 vs. Vehicle
IL-1α	26 ± 3.14	6.91 ± 0.47	7.75 ± 0.75	ns	ns
IL-1β	24.04 ± 4.92	10.42 ± 2.11	15.93 ± 2.63	*	ns
IL-2	4.49 ± 1.10	nd	nd	n/a	n/a
IL-5	2.96 ± 0.37	2.85 ± 1.03	3.52 ± 0.60	ns	ns
IL-9	99.56 ± 12.57	132.10 ± 24.21	116.00 ± 19.28	ns	ns
IL-10	3.89 ± 0.25	3.10 ± 0.84	3.19 ± 0.47	ns	ns
IL-12p40	11.27 ± 1.07	10.15 ± 1.13	10.20 ± 0.49	ns	ns
IL-12p70	8.93 ± 1.17	4.62 ± 1.19	5.74 ± 0.90	*	ns
IL-13	98.20 ± 14.41	76.03 ± 14.45	98.11 ± 14.37	ns	ns
IL-17A	7.94 ± 1.27	3.29 ± 0.66	4.41 ± 0.90	**	*
Eotaxin	264.20 ± 30.39	135.20 ± 49.53	237.40 ± 34.22	ns	ns
G-CSF	4.62 ± 0.95	2.57 ± 0.54	3.89 ± 0.21	*	ns
GM-CSF	29.95 ± 2.66	nd	28.82 ± 10.48	n/a	ns
IFN-γ	5.22 ± 0.71	3.90 ± 1.03	6.13 ± 0.79	ns	ns
KC	13.92 ± 2.64	7.91 ± 0.69	11.71 ± 1.47	*	ns
MCP-1	84.93 ± 9.12	70.74 ± 12.04	67.41 ± 7.78	ns	ns
MIP-1β	16.94 ± 1.99	15.80 ± 2.38	16.04 ± 2.05	ns	ns
RANTES	6.95 ± 0.59	6.86 ± 1.04	8.47 ± 1.07	ns	ns
TNF-α	6.59 ± 0.33	4.44 ± 0.63	7.55 ± 0.81	ns	ns

Data are expressed as mean ± SEM. Statistical significance is indicated: * *p* < 0.05, ** *p* < 0.01. ns: not significant; nd: not detectable; n/a: not applicable. GM-CSF, granulocyte-macrophage colony-stimulating factor; KC, keratinocyte chemoattractant, TNF-α, tumor necrosis factor alpha.

## Supplementary Materials

Supplementary materials can be found at www.mdpi.com/1422-0067/19/1/207/s1.
