# Supplementary material for: Synthesized Heparan Sulfate Competitors Attenuate Pseudomonas aeruginosa Lung Infection
Source: Int J Mol Sci. 2018 Jan 9;19(1):207. doi: 10.3390/ijms19010207 (PMC5796156; doi:10.3390/ijms19010207)
Supplement: Supplementary file 1 [file ijms-19-00207-s001.pdf]

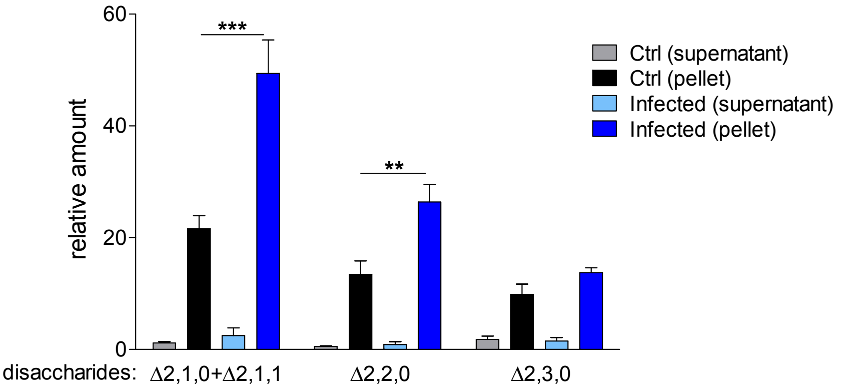

(**a**)

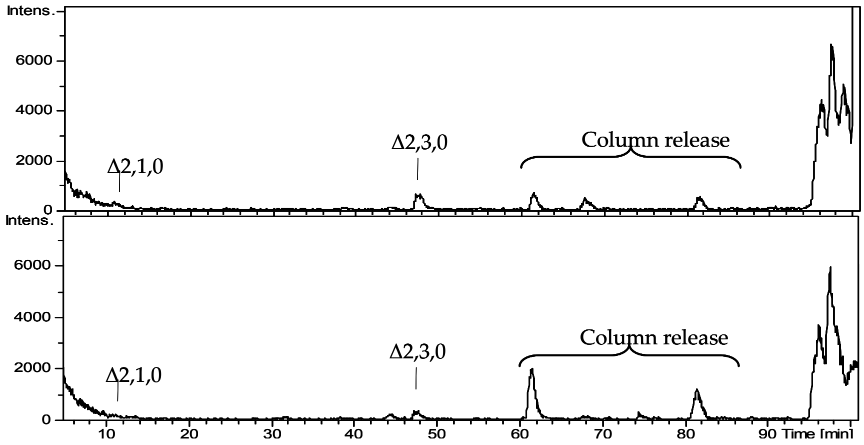

(**b**)

Figure S1. Disaccharide products of the digestion, with Heparinase lyases, of murine lung after chronic *P. aeruginosa* infection. C57Bl/6NcrIBR mice were intratracheally injected with 1–2 × 10^6 colony forming units (CFUs) of *P. aeruginosa* isolate AA43 embedded in agar-beads (Infected) or with sterile agar-beads (Ctrl). After 28 days, lungs were perfused, recovered, homogenized and separated into pellets and supernatants. After removal of proteins, lipids and DNA, the presence of GAGs was verified by NMR. Samples were digested with a cocktail of heparin lyases to selectively degrade heparin/HS and recovered digestion products were desalted and finally analyzed by HPLC-MS. (**a**) The graph shows the percentage of each disaccharide species relative to the disaccharide moiety in each sample. 100% is considered the sum of peak areas of one whole lung from infected mouse lungs containing the highest amount of disaccharides. The data are pooled from at least two independent experiments (n=6-15). Data are the mean of at least three samples per type which have been processed independently. Statistical significance is indicated: ** $p < 0.01$; *** $p < 0.001$; (**b**) Base Peak Chromatogram of digestion products from the supernatant of one control uninfected (**upper** panel) and one infected (**lower** panel) lung homogenate from C57Bl/6NcrIBR mice. The Δ symbol indicates the unsaturation introduced by enzymes, followed by the number of monomers, number of sulfates and acetyls.

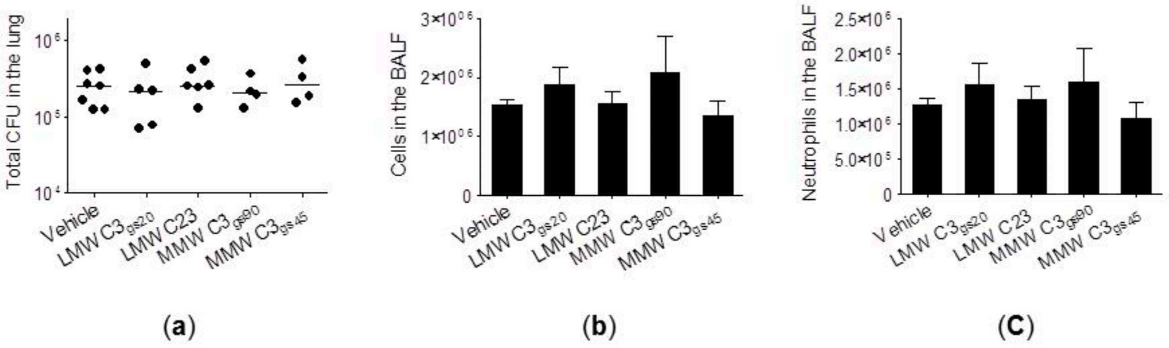

Figure S2. Modulation of inflammation by synthesized HS competitors in a mouse model of acute *P. aeruginosa* lung infection. C57Bl/6NcrlBR mice were intratracheally injected with 1 × 10^6 CFU of the highly virulent *P. aeruginosa* isolate AA2. Mice were subcutaneously treated with the two synthesized HS competitors (30 mg/kg) or their vehicle two hours before and two hours after the challenge and sacrificed 6 h post-infection. BALF and lung were recovered. (**a**) Total CFUs in the lungs were evaluated; (**b**) Total cell and (**c**) neutrophil recruitment was analyzed in BALF. The data are pooled from two independent experiments (*n* = 4–7). CFUs in individual mice are represented as dots and horizontal lines represent median values. Cells are represented as mean ± SEM.

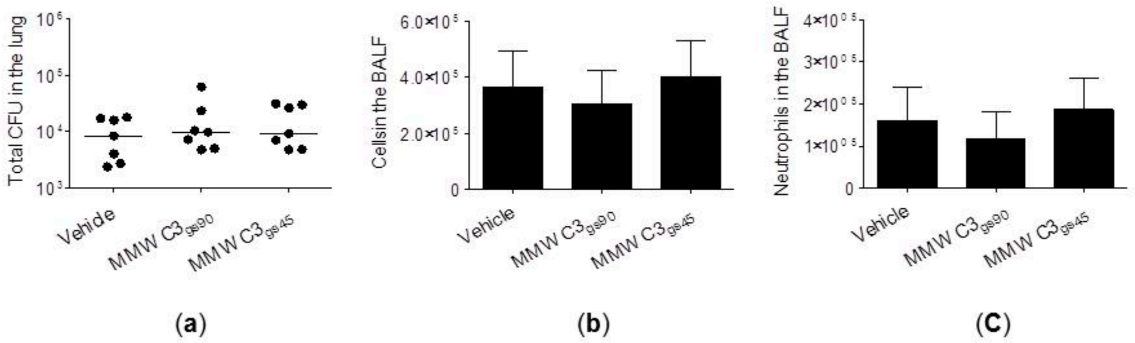

Figure S3. Modulation of the host response by synthesized HS competitors in a mouse model of long-term chronic *P. aeruginosa* lung infection (28 days). C57Bl/6NcrlBR mice were intratracheally injected with 1–2 × 10^6 CFU of the *P. aeruginosa* isolate AA43 embedded in agar-beads. Mice were treated subcutaneously with synthesized HS competitors (30 mg/kg) or vehicle every day starting from ten days post-infection. At the sacrifice, BALF and lung were recovered. (**a**) Total CFUs in the lungs were evaluated; (**b**) Total cell and (**c**) neutrophil recruitment was analyzed in BALF. The data are pooled from at least two independent experiments (*n* = 23–26). CFUs in individual mice are represented as dots and horizontal lines represent median values. Cells are represented as mean ± SEM.

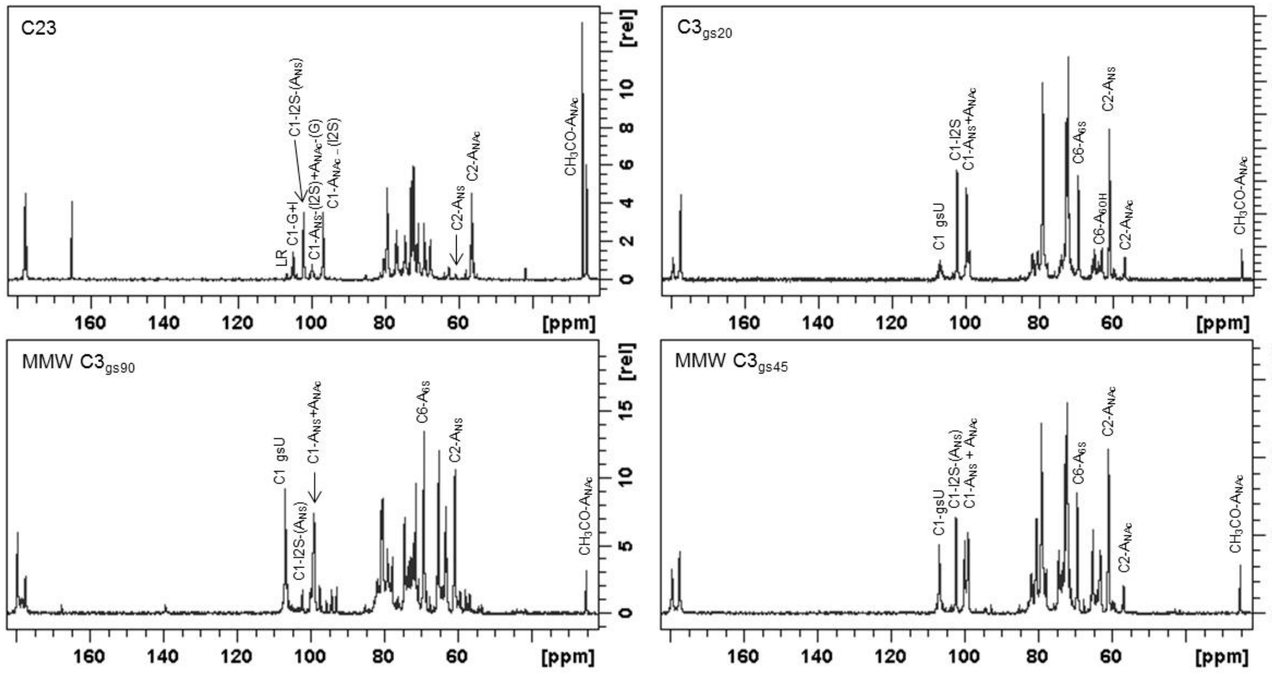

(**a**)

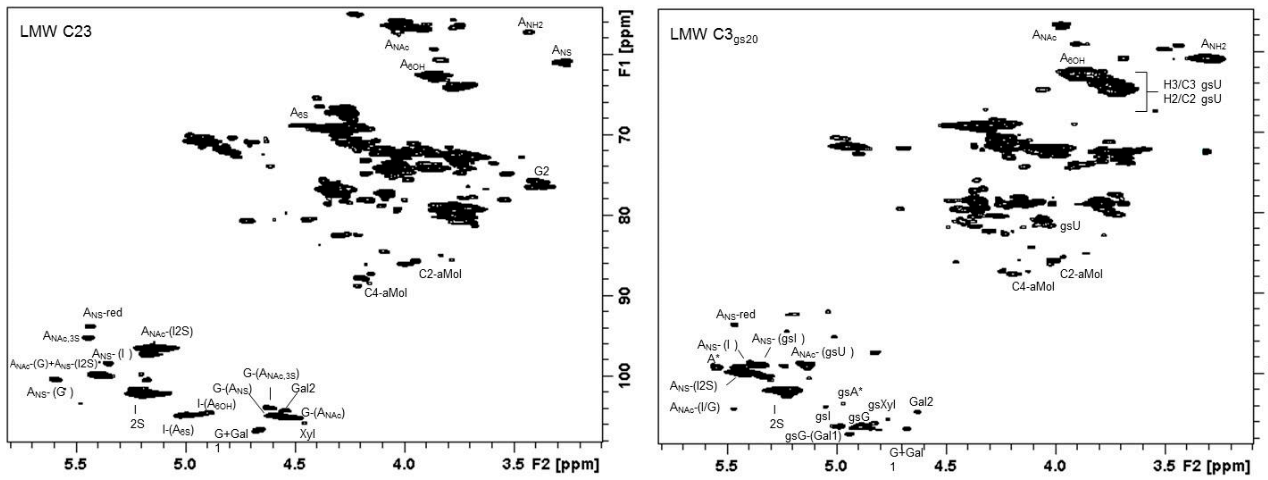

(**b**)

Figure S4. (**a**) ^{13}C-NMR spectra of compounds C23, C3$_{gs20}$, MMW C3$_{gs90}$ and MMW C3$_{gs45}$. Attribution of the main signals is indicated as follows: I2S—2-O-sulfated iduronic acid, I/G—non-sulfated iduronic/glucuronic acid, gsU—glycol-split uronic acid/xylose, ANS/ANAc—*N*-sulfated/*N*-acetylated glucosamine, A6S—6-*O*-sulfated glucosamine, LR—linkage region; (**b**) HSQC-NMR spectra of compounds LMW C3$_{gs20}$ and LMW C23, obtained by deaminative reduction of pig mucosa heparin (PMH) followed by glycol-splitting (LMW C3$_{gs20}$) or *N*-desulfation and *N*-acetylation (LMW C23). Attribution of the main signals is indicated as follows: I2S—2-*O*-sulfated iduronic acid, I/G—non-sulfated iduronic/glucuronic acid, gsI/gsG/gsXyl—glycol-split iduronic/glucuronic acid/xylose, ANS/ANAc—*N*-sulfated/*N*-acetylated glucosamine, A6S—6-*O*-sulfated glucosamine, A*—*N*,6-*O*,3-*O*-sulfated glucosamine, Xyl—xylose, Gal—galactose, aMol—anhydromannithol, introduced by the reductive deamination.

Supplementary Materials and Methods

Mouse model. Mice were maintained in pathogen free conditions and tested as previously described [1,2]. For infection experiments, mice were anesthetized by an intraperitoneal injection of a solution of Avertin (2,2,2-tribromethanol, 97%) in 0.9% NaCl and administered at a volume of 0.015 mL/g body weight. Mice were placed in supine position. The trachea was directly visualised by ventral midline, exposed and intubated with a sterile, flexible 22-g cannula attached to a 1 mL

syringe. An inoculum of 60 μL of planktonic bacteria or 50 μL of agar bead suspension was implanted via the cannula into the lung and all lobes were inoculated. After inoculation, all incisions were closed by suture. All mice were maintained under specific pathogen-free conditions in sterile cages which were put into a ventilated isolator. Fluorescent lights were cycled 12 h on, 12 h off, and ambient temperature (23 ± 1 °C) and relative humidity (40–60%) were regulated. Mice were fed with standard rodent autoclaved chow and autoclaved tap water.

Mice were monitored twice per day for the parameters vocalisation, piloerection, attitude, locomotion, breathing, curiosity, nasal secretion, grooming and dehydration. Mice that lost >20% body weight and had evidence of severe clinical disease, such as scruffy coat, inactivity, loss of appetite, poor locomotion, or painful posture, were sacrificed before the termination of the experiments with an overdose of carbon dioxide.

Bronchoalveolar lavage fluid (BALF) was extracted with a 22-gauge venous catheter by washing the lungs with RPMI-1640 (Euroclone) with protease inhibitors (Complete tablets, Roche Diagnostic and PMSF, Sigma). Total cells present in the BALF were counted, and a differential cell count was performed on cytospins stained with Diff Quick (Dade, Biomap, Italy). BALF was serially diluted and plated on trypticase soy agar (TSA).

Lungs were excised aseptically and homogenized in 2 mL PBS using the homogenizer gentleMACS™ Octo Dissociator and dilutions spotted onto TSA plates and CFUs determined after 24 h growth at 37 °C. Recovery of >1000 CFU of *P. aeruginosa* from cultures of lung was considered as evidence of chronic infection.

Animal studies were conducted according to protocols approved by San Raffaele Scientific Institute (Milan, Italy) Institutional Animal Care and Use Committee (IACUC) and adhered strictly to the Italian Ministry of Health guidelines for the use and care of experimental animals.

HS/heparin analysis in murine lungs. Ethyl ether, Proteinase K and DNase I were from Sigma Aldrich, USA. Syringe filters (0.22 μm and 0.45 μm) and Amicon ULTRA ultrafiltration filters were from Millipore, USA. Samples were kept in cold acetone (Sigma Aldrich, USA) overnight at 4 °C, then defatted by washing with 2:1 and 1:1 chloroform/methanol solutions (Sigma Aldrich, USA) and filtered by 3 μm cut-off to discard solvents. After inactivation of Proteinase K by boiling for 10 min, the temperature was set to 37 °C, $MgCl_2$ was added at a final concentration of 2 mM prior to the addition of DNase I.

Desalting of digestion products was conducted on a G-10 column (h 25 cm, Ø 1.5 cm) by 10% EtOH elution and collection of 400 μL fractions, followed by detection at 210/232 nm (Cary50 UV, Varian). The LC system (Dionex Ultimate 3000, Dionex) is equipped with degassing system, pump, autosampler and UV-detector and coupled with an ESI-Q-TOF mass-spectrometer (micrOTOFq, Bruker Daltonics, Germany). The MS spectrometric conditions were as follows: ESI in negative ion mode, drying gas temperature +180 °C, drying gas flow-rate 7.0 L/min, nebulizer pressure 0.9 bar; and capillary voltage +3.2 kV. The mass spectra of the oligosaccharides were acquired in a scan mode (*m*/*z* scan range 200–2000). Calibration of the mass spectrometer was obtained by using an ES tuning mix solution acetonitrile solution (Agilent Technologies, Santa Clara, CA, USA) according to a standard procedure.

HS competitors synthesis and characterization. Reductive deamination on PMH was conducted as follows: 33 mg of $NaNO_2$ (Sigma Aldrich, USA) were added to a 60 mg/mL PMH aqueous solution at 18 °C and the pH was adjusted to 2.4. After 30 min, pH was reported to neutrality and 90 mg of NaBH4 (Sigma Aldrich, USA) were added and the pH was adjusted to 3 for 10 min followed by neutralization. Samples were purified by ethanol precipitation using a NaOAc-saturated EtOH solution at 32%, then centrifuged for 1 h (Avanti Beckman). After discarding of the supernatant, the procedure was repeated with NaOAc-saturated 53% and 99% EtOH solutions followed by acetone washing. Recovered pellet was desalted onto a TSK column (h 1 m, Ø 5 cm, TOSOH Toyopearl) and purity of compounds was verified by NMR.

Evaluation of *P. aeruginosa* biofilm formation. Biofilm production was evaluated using the method of staining with crystal violet [3]. Wells of flat-bottom polystyrene microplates (Corning, 3590) were filled with 200 μL of a tryptic soy broth culture of *P. aeruginosa* strain AA43 grown at mid-exponential phase with 1% of glucose. Wells were treated with vehicle, or $C3_{gs20}$ or C23 at three

different doses (10 μg/mL, 1 μg/mL and 0.1 μg/mL) and incubated at 37 °C. After 24 h the supernatant of each well was gently transferred into another microplate and OD_{600} was measured. The plate was rinsed three times and air-dried for 30 min. 200 μL of 1% crystal violet was then added to each well at room temperature to stain the biofilm. After 30 min, wells were rinsed three times or until crystal violet excess was completely removed. When the microplate was dry, 200 μL/well of 95% ETOH was added to dissolve the bound dye and left for 30 min in ice. Then, absorbance was measured at OD_{600} to quantify biofilm production.

References

1. Cigana, C.; Lore, N.I.; Riva, C.; de Fino, I.; Spagnuolo, L.; Sipione, B.; Rossi, G.; Nonis, A.; Cabrini, G.; Bragonzi, A. Tracking the immunopathological response to *Pseudomonas aeruginosa* during respiratory infections. *Sci. Rep.* **2016**, *6*, 21465.
2. Kukavica-Ibrulj, I.; Facchini, M.; Cigana, C.; Levesque, R.C.; Bragonzi, A. Assessing *Pseudomonas aeruginosa* virulence and the host response using murine models of acute and chronic lung infection. *Methods Mol. Biol.* **2014**, *1149*, 757–771.
3. Baldan, R.; Cigana, C.; Testa, F.; Bianconi, I.; de Simone, M.; Pellin, D.; di Serio, C.; Bragonzi, A.; Cirillo, D.M. Adaptation of *Pseudomonas aeruginosa* in Cystic Fibrosis airways influences virulence of Staphylococcus aureus in vitro and murine models of co-infection. *PLoS ONE* **2014**, *9*, e89614.
